# Supplementary material for: Germline Variants in 32 Cancer-Related Genes among 700 Chinese Breast Cancer Patients by Next-Generation Sequencing: A Clinic-Based, Observational Study
Source: Int J Mol Sci. 2022 Sep 24;23(19):11266. doi: 10.3390/ijms231911266 (PMC9570072; doi:10.3390/ijms231911266)
Supplement: Supplementary file 1 [file ijms-23-11266-s001.zip › Figure S1.pdf]

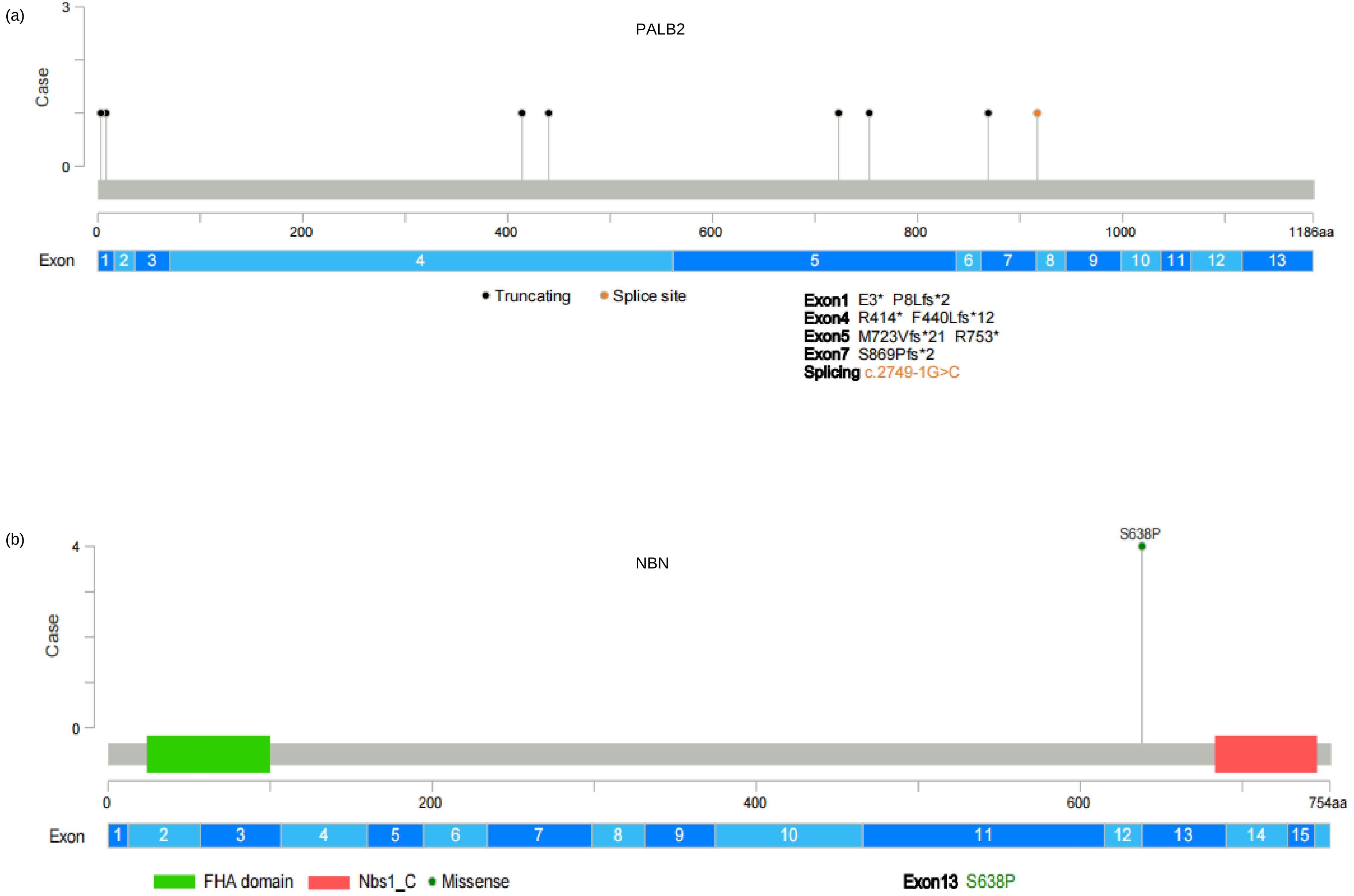
(a)
PALB2
Case
3
0
0
200
400
600
800
1000
1186aa
Exon
1 2 3 4 5 6 7 8 9 10 11 12 13
Truncating
Splice site
Exon1 E3* P8Lfs*2
Exon4 R414* F440Lfs*12
Exon5 M723Vfs*21 R753*
Exon7 S869Pfs*2
Splicing c.2749-1G>C
(b)
NBN
S638P
Case
4
0
0
200
400
600
754aa
Exon
1 2 3 4 5 6 7 8 9 10 11 12 13 14 15
FHA domain
Nbs1_C
Missense
Exon13 S638P

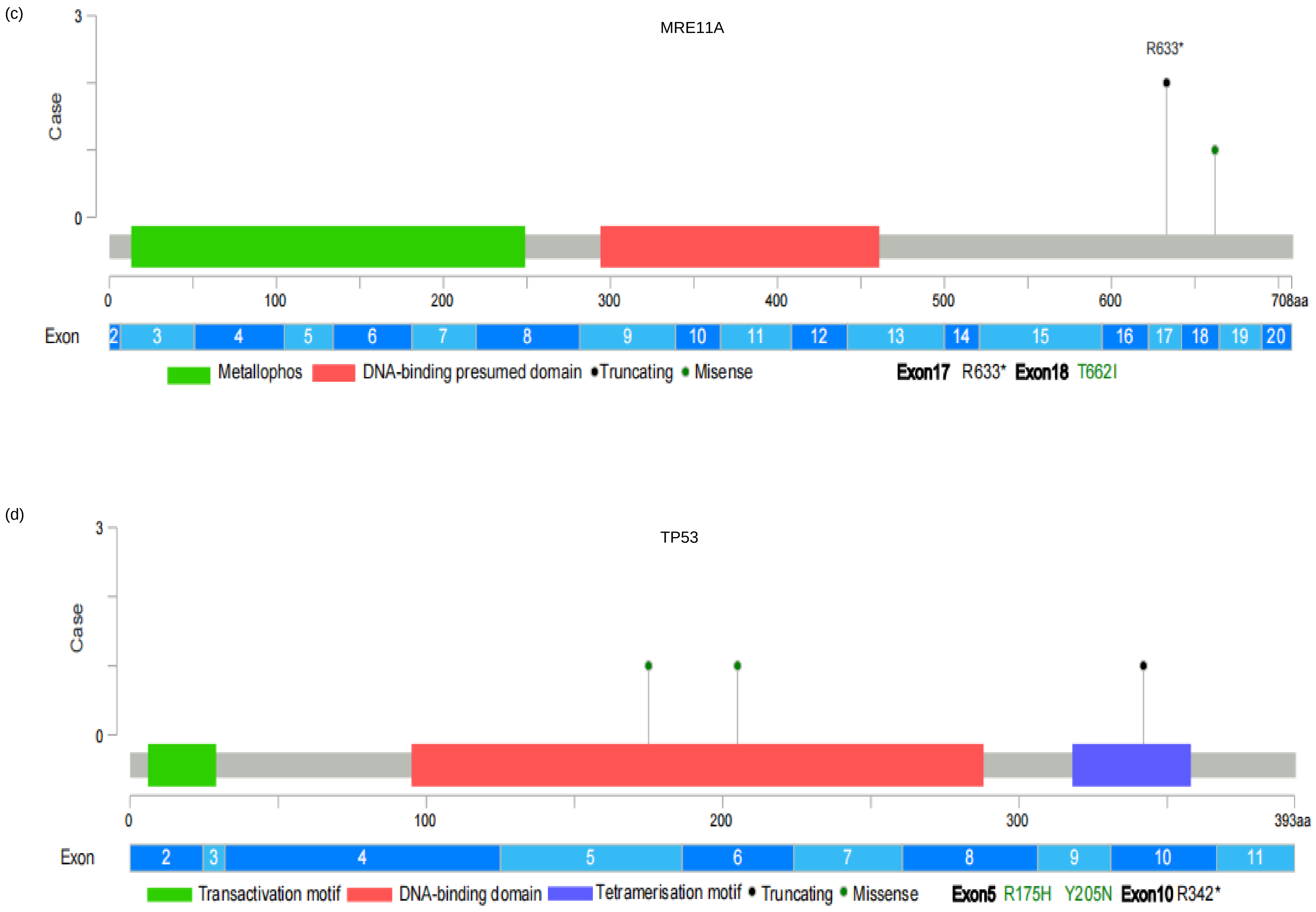
(c)
MRE11A
Case
3
0
R633*
0
100
200
300
400
500
600
708aa
Exon
2 3 4 5 6 7 8 9 10 11 12 13 14 15 16 17 18 19 20
Metallophos
DNA-binding presumed domain
Truncating
Misense
Exon17 R633* Exon18 T662I
(d)
TP53
Case
3
0
0
100
200
300
393aa
Exon
2 3 4 5 6 7 8 9 10 11
Transactivation motif
DNA-binding domain
Tetramerisation motif
Truncating
Missense
Exon5 R175H Y205N Exon10 R342*

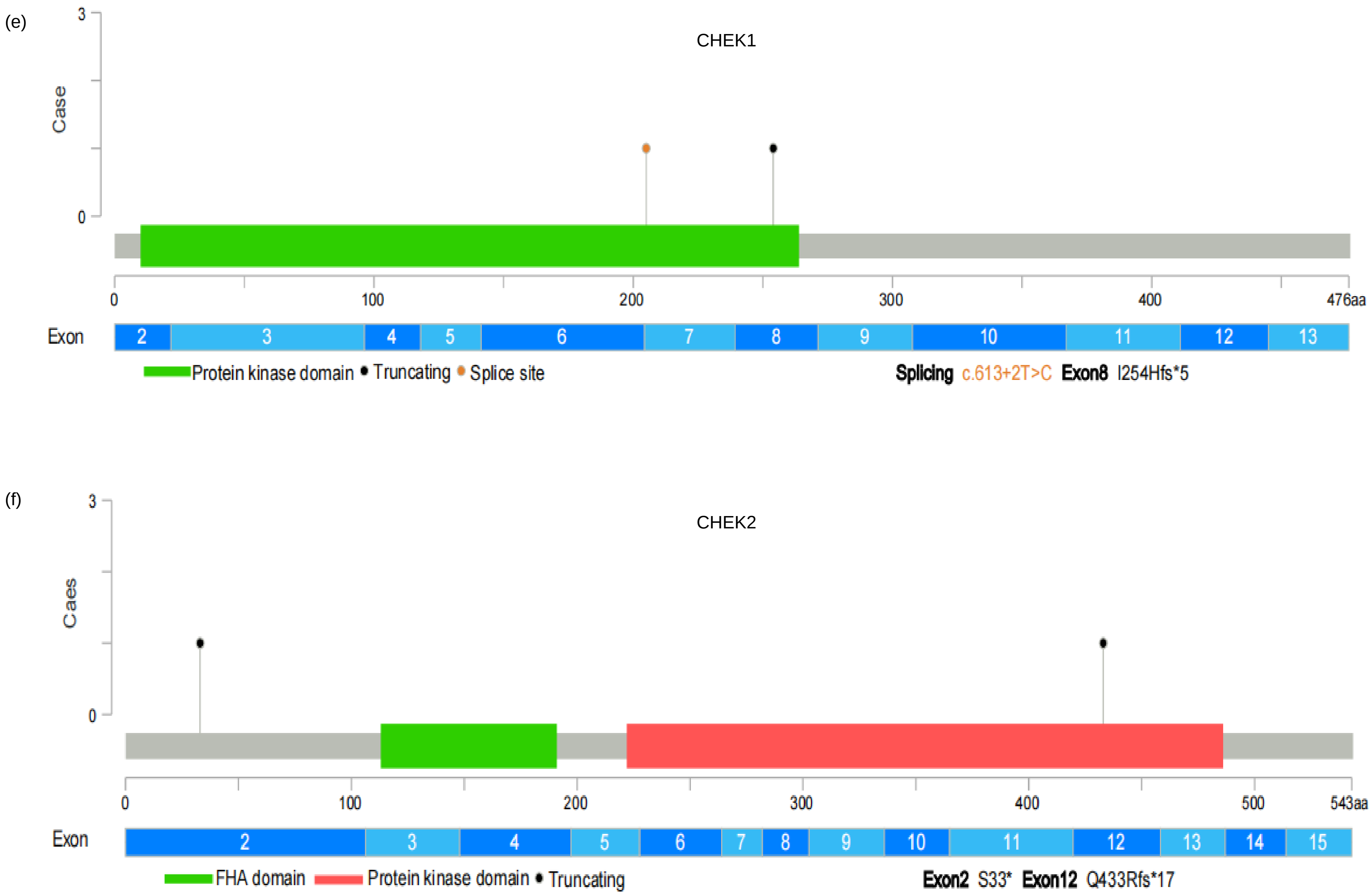

(e)
CHEK1
Case
3
0
0
100
200
300
400
476aa
Exon
2 3 4 5 6 7 8 9 10 11 12 13
Protein kinase domain
Truncating
Splice site
Splicing c.613+2T>C Exon8 I254Hfs*5
(f)
CHEK2
Caes
3
0
0
100
200
300
400
500
543aa
Exon
2 3 4 5 6 7 8 9 10 11 12 13 14 15
FHA domain
Protein kinase domain
Truncating
Exon2 S33* Exon12 Q433Rfs*17

(g)

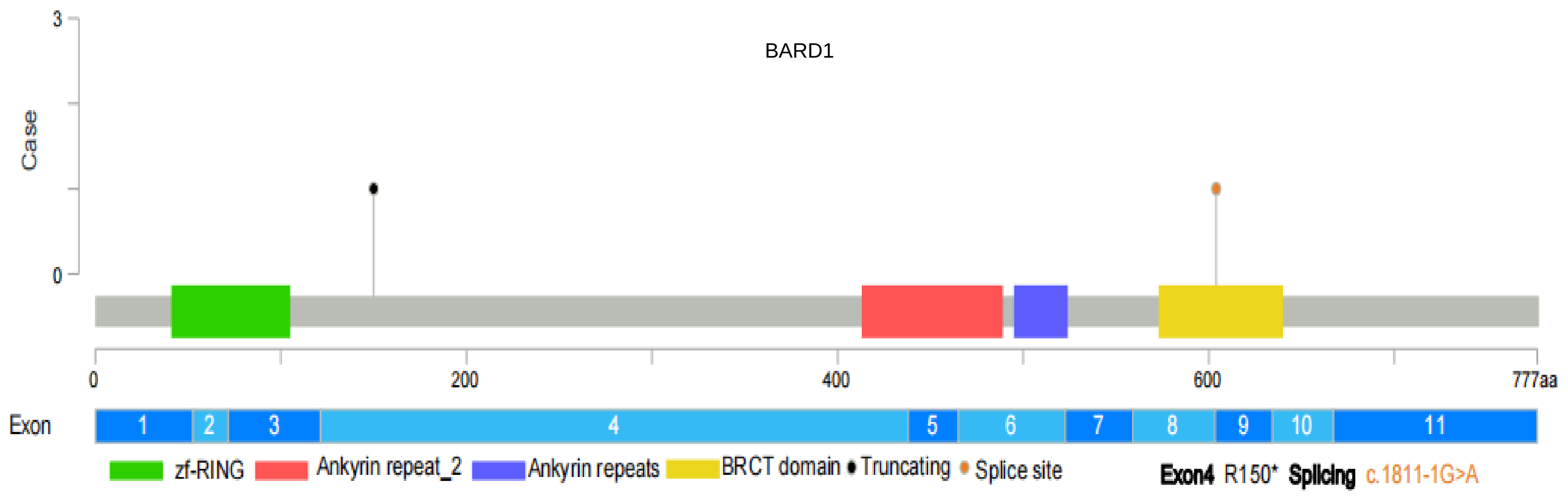
BARD1
Case
3
0
0
200
400
600
777aa
Exon
1 2 3 4 5 6 7 8 9 10 11
zf-RING
Ankyrin repeat_2
Ankyrin repeats
BRCT domain
Truncating
Splice site
Exon4 R150* Splicing c.1811-1G>A

(h)

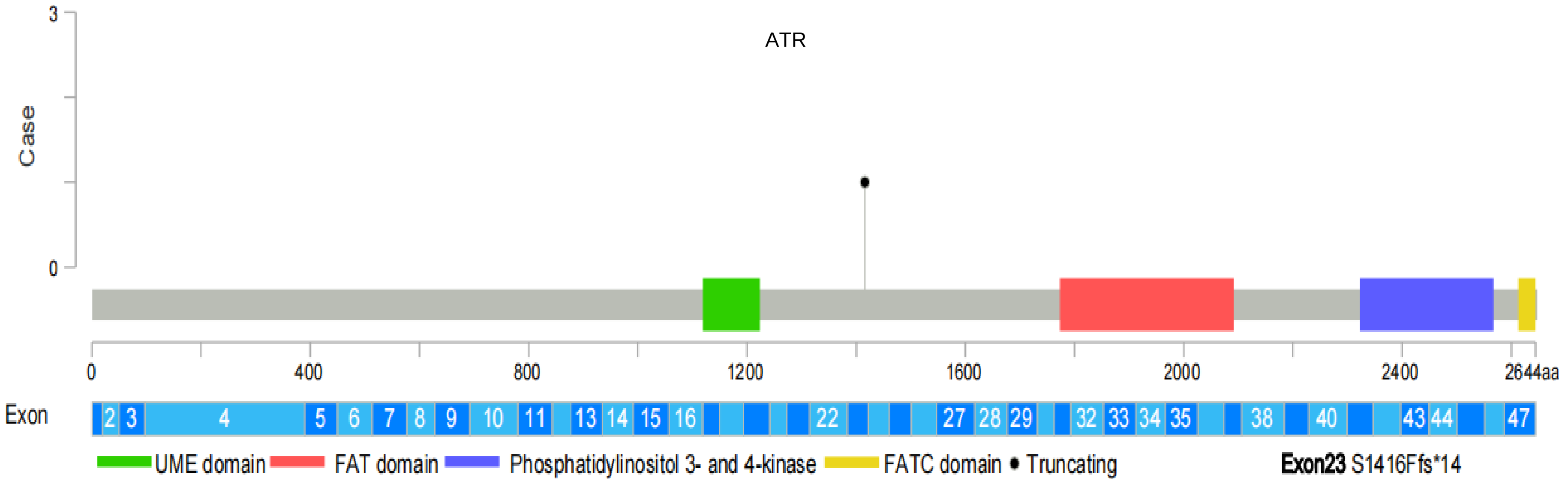
ATR
Case
3
0
0
400
800
1200
1600
2000
2400
2644aa
Exon
2 3 4 5 6 7 8 9 10 11 13 14 15 16 22 27 28 29 32 33 34 35 38 40 43 44 47
UME domain
FAT domain
Phosphatidylinositol 3- and 4-kinase
FATC domain
Truncating
Exon23 S1416Ffs*14

(i)

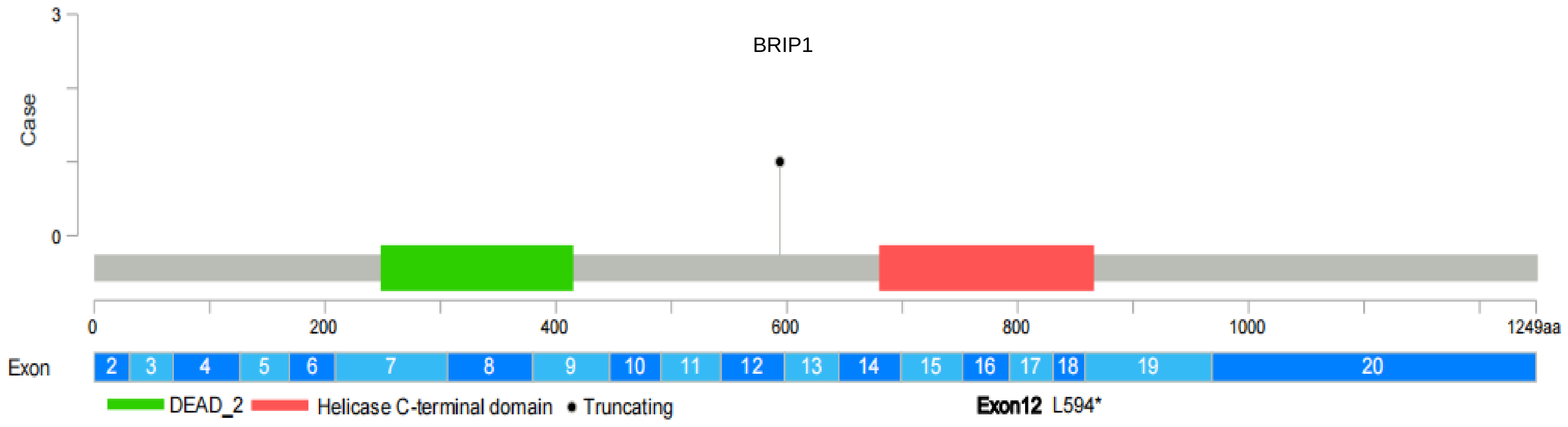
BRIP1
Case
3
0
0
200
400
600
800
1000
1249aa
Exon
2 3 4 5 6 7 8 9 10 11 12 13 14 15 16 17 18 19 20
DEAD_2
Helicase C-terminal domain
Truncating
Exon12 L594*

(j)

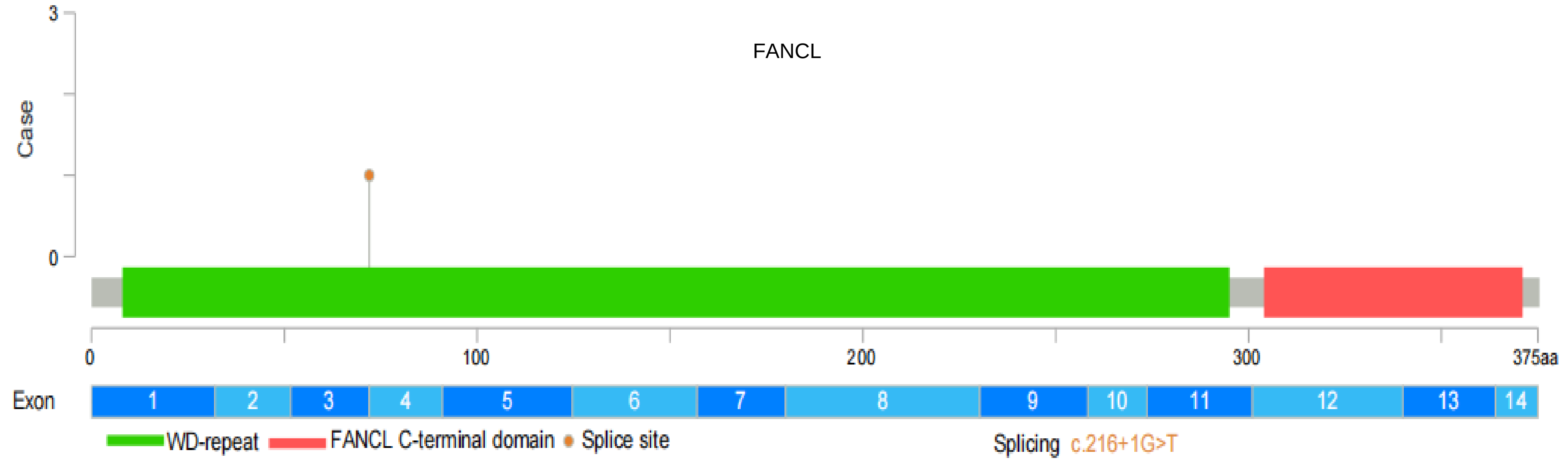
FANCL
Case
3
0
0
100
200
300
375aa
Exon
1 2 3 4 5 6 7 8 9 10 11 12 13 14
WD-repeat
FANCL C-terminal domain
Splice site
Splicing c.216+1G>T

(k)

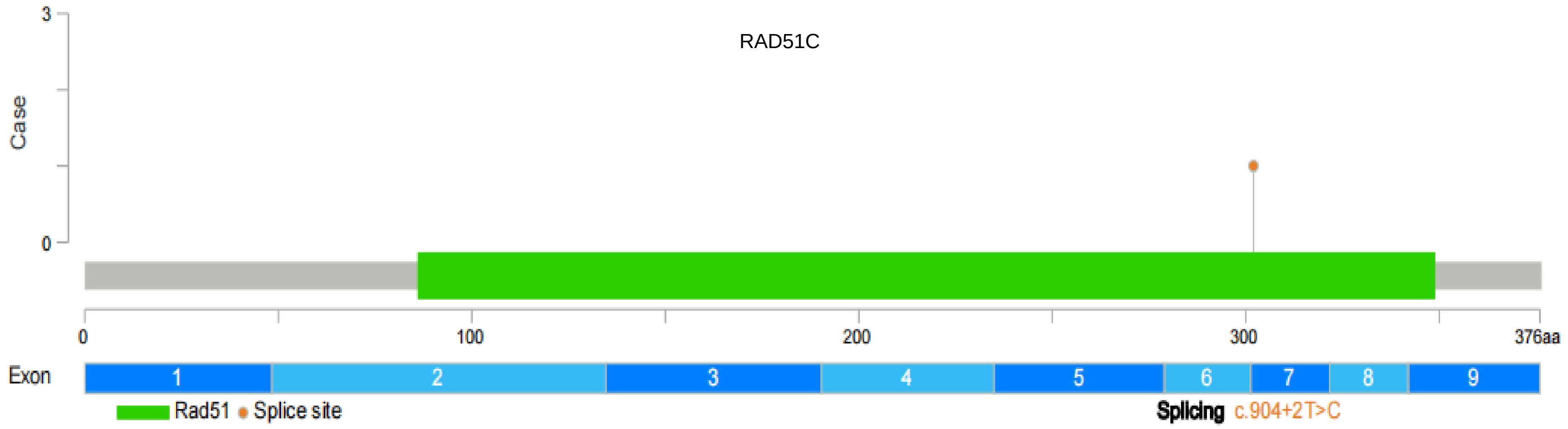

RAD51C
Case
3
0
0
100
200
300
376aa
Exon
1
2
3
4
5
6
7
8
9
Rad51
Splice site
Splicing c.904+2T>C


(l)

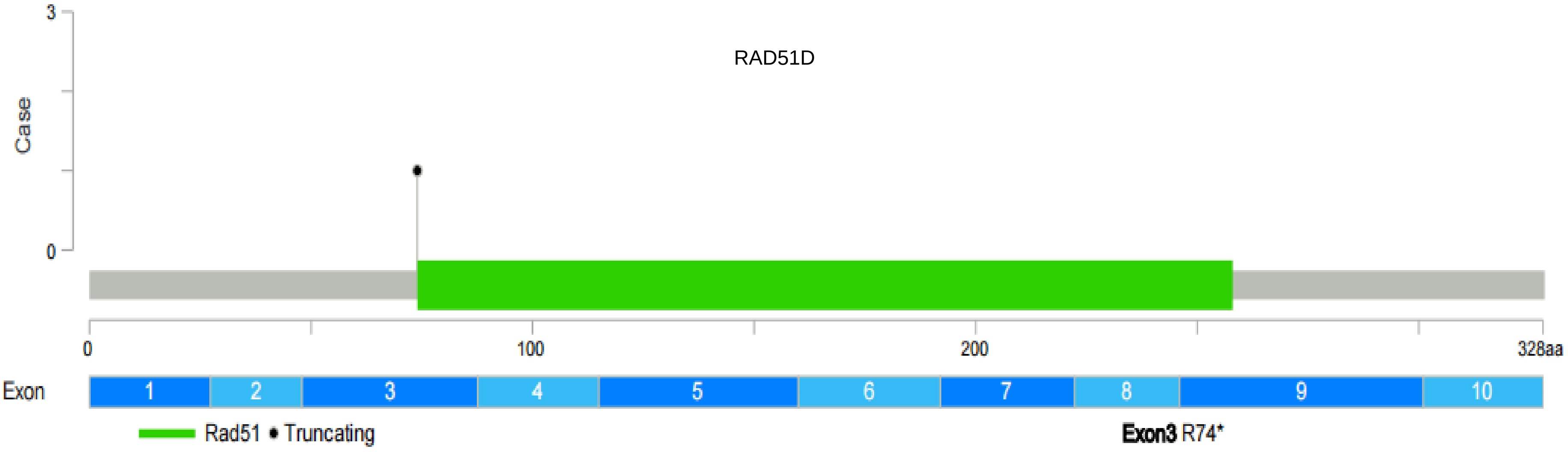

RAD51D
Case
3
0
0
100
200
328aa
Exon
1
2
3
4
5
6
7
8
9
10
Rad51
Truncating
Exon3 R74*

(m)

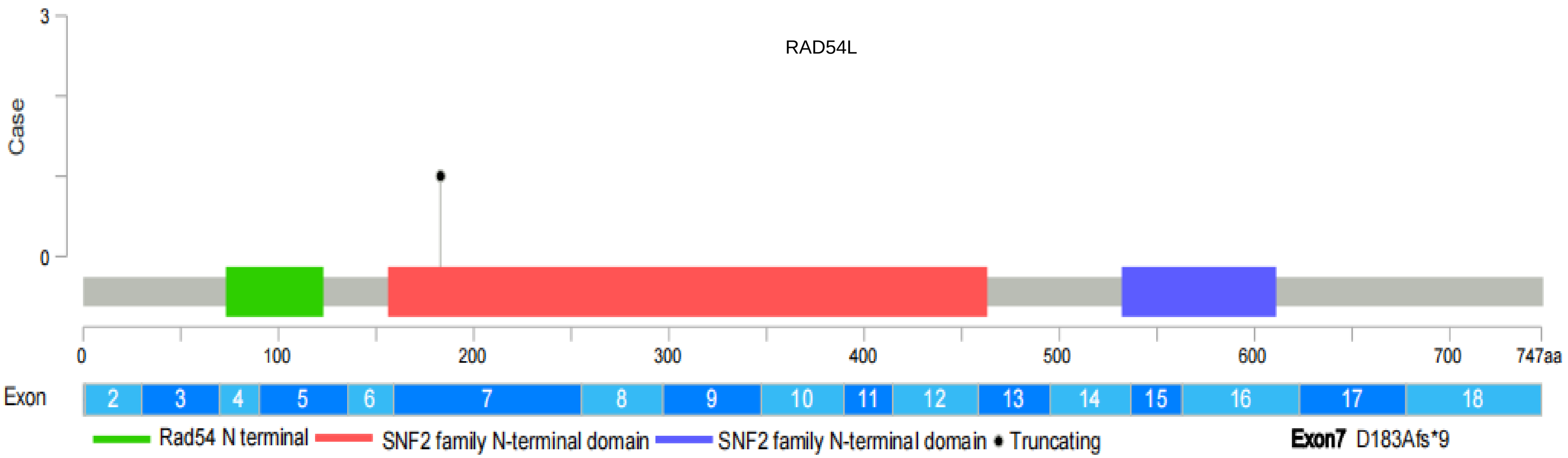


**Figure S1:** Locations of variations in the non-BRCA1/2 proteins. (a) The variations in PALB2 protein. (b) The variations in NBN protein. (c) The variations in MRE11A protein. (d) The variations in TP53 protein. (e) The variations in CHEK1 protein. (f) The variations in CHEK2 protein. (g) The variations in BARD1 protein. (h) The variations in ATR protein. (i) The variations in BRIP1 protein. (j) The variations in FANCL protein. (k) The variations in RAD51C protein. (l) The variations in RAD51D protein. (m) The variations in RAD54L protein.
