# Supplementary material for: Germline Variants in 32 Cancer-Related Genes among 700 Chinese Breast Cancer Patients by Next-Generation Sequencing: A Clinic-Based, Observational Study
Source: Int J Mol Sci. 2022 Sep 24;23(19):11266. doi: 10.3390/ijms231911266 (PMC9570072; doi:10.3390/ijms231911266)
Supplement: Supplementary file 1 [file ijms-23-11266-s001.zip › Table S1.pdf]

Table S1. Distribution of deleterious germline variants according to the type of cancer and genetic relationship in breast cancer patients with a positive family history (n = 213).

Family History	No.	Panel-gene variants			HRR-gene variants			*BRCA1/2* variants		
		Carrier n = 38	Non-carrier n = 175	p	Carrier n = 36	Non-carrier n = 177	p	Carrier n = 30	Non-carrier n = 183	p
Breast cancer (%)										
Yes	112	25 (65.8)	87 (49.7)	0.105	23 (63.9)	89 (50.3)	0.191	20 (66.7)	92 (50.3)	0.142
No	101	13 (34.2)	88 (50.3)		13 (36.1)	88 (49.7)		10 (33.3)	91 (49.7)	
BRCA-related cancer[1] (%)										
Yes	127	28 (73.7)	99 (56.6)	0.077	26 (72.2)	101 (57.1)	0.133	23 (76.7)	104 (56.8)	0.064
No	86	10 (26.3)	76 (43.4)		10 (27.8)	76 (42.9)		7 (23.3)	79 (43.2)	
Gastrointestinal cancer (%)										
Yes	50	8 (21.1)	42 (24.0)	0.859	7 (19.4)	43 (24.3)	0.682	7 (23.3)	43 (23.5)	1.000
No	163	30 (78.9)	133 (76.0)		29 (80.6)	134 (75.7)		23 (76.7)	140 (76.5)	
Hepatobiliary cancer (%)										
Yes	17	3 (7.9)	14 (8.0)	1.000	3 (8.3)	14 (7.9)	1.000	3 (10.0)	14 (7.7)	0.939
No	196	35 (92.1)	161 (92.0)		33 (91.7)	163 (92.1)		27 (90.0)	169 (92.3)	
Lung cancer (%)										
Yes	41	8 (21.1)	33 (18.9)	0.933	8 (22.2)	33 (18.6)	0.791	6 (20.0)	35 (19.1)	1.000
No	172	30 (78.9)	142 (81.1)		28 (77.8)	144 (81.4)		24 (80.0)	148 (80.9)	
Gynecological cancer[2] (%)										
Yes	9	3 (7.9)	6 (3.4)	0.426	3 (8.3)	6 (3.4)	0.374	3 (10.0)	6 (3.3)	0.228
No	204	35 (92.1)	169 (96.6)		33 (91.7)	171 (96.6)		27 (90.0)	177 (96.7)	
Urinary cancer[3] (%)										
Yes	7	0 (0.0)	7 (4.0)	0.452	0 (0.0)	7 (4.0)	0.484	0 (0.0)	7 (3.8)	0.591
No	206	38 (100.0)	168 (96.0)		36 (100.0)	170 (96.0)		30 (100.0)	176 (96.2)	
Hematologic cancer (%)										
Yes	11	1 (2.6)	10 (5.7)	0.708	1 (2.8)	10 (5.6)	0.767	1 (3.3)	10 (5.5)	0.965
No	202	37 (97.4)	165 (94.3)		35 (97.2)	167 (94.4)		29 (96.7)	173 (94.5)	
Genetic relationship (%)										
First-Degree only	115	18 (47.4)	97 (55.4)	0.377	18 (50.0)	97 (54.8)	0.523	14 (46.7)	101 (55.2)	0.486
Second-Degree only	44	9 (23.7)	35 (20.0)		8 (22.2)	36 (20.3)		8 (26.7)	36 (19.7)	
Third-Degree only	7	0 (0.0)	7 (4.0)		0 (0.0)	7 (4.0)		0 (0.0)	7 (3.8)	
More than one degree	47	11 (28.9)	36 (20.6)		10 (27.8)	37 (20.9)		8 (26.7)	39 (21.3)	

[1]*BRCA*-related cancer: breast cancer, epithelial ovarian cancer (including fallopian tube cancer or peritoneal cancer), exocrine pancreatic cancer and prostate cancer.

[2]Gynecological cancer: ovarian cancer was not included.

[3]Urinary cancer: prostate cancer was not included.

Abbreviation: HRR=homologous recombination repair.
