# Supplementary material for: Germline Variants in 32 Cancer-Related Genes among 700 Chinese Breast Cancer Patients by Next-Generation Sequencing: A Clinic-Based, Observational Study
Source: Int J Mol Sci. 2022 Sep 24;23(19):11266. doi: 10.3390/ijms231911266 (PMC9570072; doi:10.3390/ijms231911266)
Supplement: Supplementary file 1 [file ijms-23-11266-s001.zip › Table S2.pdf]

Table S2. List of the 32 cancer-related genes in the multiple-gene NGS panel.

Gene	Reference sequence	Sequencing area
AR	NM_000044.3	Entire coding region
ATM[*, #]	NM_000051.3	Entire coding region
ATR[*, #]	NM_001184.3	Entire coding region
BARD1[*, #]	NM_000465.3	Entire coding region
BRCA1[*, #]	NM_007294.3	Entire coding region
BRCA2[*, #]	NM_000059.3	Entire coding region
BRIP1[*, #]	NM_032043.2	Entire coding region
CDH1[*]	NM_004360.3	Entire coding region
CDK12[*, #]	NM_016507.3	Entire coding region
CHEK1[*, #]	NM_001274.5	Entire coding region
CHEK2[*, #]	NM_007194.3	Entire coding region
ESR1	NM_001122740.1	Entire coding region
FANCA[*, #]	NM_000135.2	Entire coding region
FANCL[*, #]	NM_018062.3	Entire coding region
HDAC2	NM_001527.3	Entire coding region
HOXB13	NM_006361.5	Entire coding region
MRE11A[*, #]	NM_005591.3	Entire coding region
NBN[*, #]	NM_002485.4	Entire coding region
PALB2[*, #]	NM_024675.3	Entire coding region
PPP2R2A[#]	NM_002717.3	Entire coding region
PTEN[*]	NM_000314.4	Entire coding region
RAD51B[#]	NM_133509.3	Entire coding region
RAD51C[*, #]	NM_058216.2	Entire coding region
RAD51D[*, #]	NM_133629.2	Entire coding region
RAD54L[*, #]	NM_001142548.1	Entire coding region
STK11[*]	NM_000455.4	Entire coding region
TP53[*]	NM_000546.5	Entire coding region
BRAF[*]	NM_004333.4	Hot-spot (exon 11/12/15/18)
ERBB2	NM_004448.3	Hot-spot (exon 1/3/8/9/11/16-21/23/25/27)
KRAS[*]	NM_033360.3	Hot-spot (exon 2/3/4)
NRAS[*]	NM_002524.4	Hot-spot (exon 2/3/4)
PIK3CA	NM_006218.2	Hot-spot (exon 2/5/6/8/10/14/21)

[*] cancer-susceptibility gene

[#] HRR (homologous recombination repair)-related gene
